# Transmembrane AMPA receptor regulatory proteins and AMPA receptor function in the cerebellum

**DOI:** 10.1016/j.neuroscience.2009.01.004

**Published:** 2009-09-01

**Authors:** I.D. Coombs, S.G. Cull-Candy

**Affiliations:** Department of Neuroscience, Physiology and Pharmacology, University College London, Gower Street, London WC1E 6BT, UK

**Keywords:** stargazin, synaptic transmission, cerebellar cells, glutamate receptors, single-channels, AMPAR, AMPA receptor, BG, Bergmann glia, BiP, Ig binding protein, CI, calcium impermeable, CP, calcium permeable, ER, endoplasmic reticulum, GC, granule cell, nPIST, neuronal isoform of protein-interacting specifically with TC10, PF-SC, parallel fibre–stellate cell, TARP, transmembrane AMPA receptor regulatory protein, TGN, trans-Golgi network, VDCC, voltage-dependent calcium channel

## Abstract

Heterogeneity among AMPA receptor (AMPAR) subtypes is thought to be one of the key postsynaptic factors giving rise to diversity in excitatory synaptic signaling in the CNS. Recently, compelling evidence has emerged that ancillary AMPAR subunits—the so-called transmembrane AMPA receptor regulatory proteins (TARPs)—also play a vital role in influencing the variety of postsynaptic signaling. This TARP family of molecules controls both trafficking and functional properties of AMPARs at most, if not all, excitatory central synapses. Furthermore, individual TARPs differ in their effects on the biophysical and pharmacological properties of AMPARs. The critical importance of TARPs in synaptic transmission was first revealed in experiments on cerebellar granule cells from *stargazer* mice. These lack the prototypic TARP stargazin, present in granule cells from wild-type animals, and consequently lack synaptic transmission at the mossy fibre-to-granule cell synapse. Subsequent work has identified many other members of the stargazin family which act as functional TARPs. It has also provided valuable information about specific TARPs present in many central neurons. Because much of the initial work on TARPs was carried out on stargazer granule cells, the important functional properties of TARPs present throughout the cerebellum have received particular attention. Here we discuss some of these recent findings in relation to the main TARPs and the AMPAR subunits identified in cerebellar neurons and glia.

AMPA receptors (AMPAR) underlie a majority of fast excitatory transmission in the mammalian brain. The subunits that form these receptors are differentially expressed throughout the CNS, and their distribution changes during development. Four main AMPAR subunits have been identified, each of which exists in several distinct splice variants. This diversity is further increased by RNA editing of the GluR2 subunit at the “Q/R-site” in its pore-lining region. Until recently, heterogeneity in AMPAR subunit properties was thought to be the main postsynaptic factor conferring diversity on excitatory signaling and information processing at central synapses. However, with the discovery of stargazin and the family of related transmembrane AMPA receptor regulatory proteins (TARPs) (Letts et al., 1998; Chen et al., 2000; Tomita et al., 2003; Priel et al., 2005; Turetsky et al., 2005), it has become increasingly clear that ancillary AMPAR proteins also shape many aspects of AMPAR function, making a vital and unexpected contribution to excitatory synaptic- and neuronal–glial signalling (Tomita et al., 2003; Nicoll et al., 2006).

TARPs modify many AMPAR properties, including their single-channel conductance (Tomita et al., 2005b; Soto et al., 2007), channel kinetics, open probability (Priel et al., 2005; Turetsky et al., 2005; Milstein et al., 2007), polyamine sensitivity (Soto et al., 2007) and interaction with agonists and antagonists (Tomita et al., 2006; Menuz et al., 2007; Kott et al., 2007). In addition, these ancillary molecules regulate the basal and activity-dependent trafficking of AMPARs (Chetkovich et al., 2002; Choi et al., 2002; Ives et al., 2004; Bedoukian et al., 2006; Bats et al., 2007). TARPs thus play a key role, not only in normal synaptic transmission, but also in neuronal development and plasticity (Nicoll et al., 2006; Osten and Stern-Bach, 2006; Ziff, 2007), and potentially in a variety of disease states.

Cerebellar neurons have been pivotal in experiments highlighting the central role of TARPs in AMPARs biology (Hashimoto et al., 1999; Chen et al., 2000). In this brief review we will describe the TARPs and AMPAR subunits known to be present in cerebellar neurons and glia, and consider their importance in controlling functional aspects of synaptic transmission and neuronal–glial interaction within the cerebellum.

## The TARP family of proteins

TARPs were first identified as proteins with homology to the γ-subunits of voltage dependent-calcium channels (VDCC). Skeletal muscle γ-1 subunits affect the voltage dependence, kinetics and pharmacology of VDCCs (Jay et al., 1990). When a neuronal γ-subunit (γ-2 or stargazin; Letts et al., 1998), was identified at the affected locus of the naturally occurring mouse mutant *stargazer*, it seemed likely that neurological defects in these mice could be ascribed to a defective neuronal VDCC. However, γ-2 and related members of this protein family, were found to produce only minor changes in calcium channel properties (Letts et al., 1998; Burgess et al., 1999, 2001; Klugbauer et al., 2000; Green et al., 2001; Rousset et al., 2001; Osten and Stern-Bach, 2006). By contrast, deficit of γ-2 has a striking effect on cerebellar synaptic transmission due to a dramatic reduction in surface AMPARs in cerebellar granule cells (GCs) (Chen et al., 1999, 2000; Hashimoto et al., 1999).

Co-expression of the stargazin (γ-2) protein with AMPAR subunits in heterologous systems markedly increases AMPAR surface expression (Chen et al., 2000; Tomita et al., 2005b; Priel et al., 2005). Furthermore, γ-2 and other recognized members of the stargazin family (γ-3, -4 and -8; Tomita et al., 2003) are capable of rescuing the surface expression of AMPARs in cultured GCs from *stargazer* mice (Tomita et al., 2003). The related molecules γ-5 and γ-7 also act as TARPs, but with different trafficking properties (Kato et al., 2008; Soto et al., 2009). They show a reduced ability to deliver AMPARs to the cell membrane, possibly because their shorter C-tails confer protein interactions that differ from those of other TARPs (Kato et al., 2007; Ferron et al., 2008). It is of note that TARP-like proteins also play an important role in regulating many invertebrate glutamate receptors, such as those in *Drosophila* and *C. elegans* (Walker et al., 2006).

## TARPs play an essential role in AMPAR trafficking

**Fig. 1:**
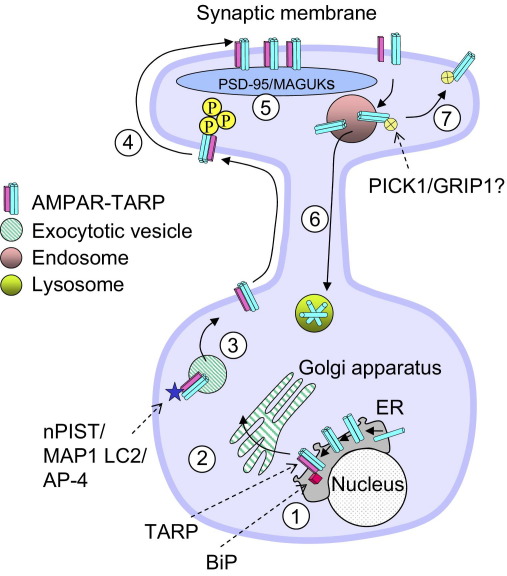
Cell trafficking of the TARP-AMPAR assembly. AMPAR biogenesis is initiated in the ER, with formation of dimers (homo or heterodimers) of GluR subunits, followed by tetramerization. TARPs interact with the AMPARs in the ER (1). Within the ER, proteins such as BiP, interact with TARPs to facilitate trafficking of the TARP–AMPAR complex through the TGN (2). In the TGN, TARPs interact with proteins including nPIST, MAP1 LC2 and AP-4, which may assist vesicular trafficking to the cell surface (3). PKC/CaMKII phosphorylate multiple sites on the TARP protein to facilitate membrane mobilization (4) and hence increase the likelihood of AMPARs accumulating at the synapse. AMPARs are immobilized in the postsynaptic membrane via interaction with MAGUKs, including PSD-95 (5). Following endocytosis, potentially triggered by agonist dependent TARP-AMPAR dissociation, the subsequent fate of the AMPARs may be degradation (6) or possibly recycling to the cell surface by other AMPAR interacting proteins such as PICK1 or GRIP1 (7).

TARPs enhance AMPAR exit from the endoplasmic reticulum (ER), promote AMPAR intracellular trafficking and cell surface expression, and preferentially stabilize TARP associated AMPARs in the postsynaptic density (Fig. 1). In expression systems, many AMPAR assemblies can exit the ER membrane and reach the cell surface in the absence of a TARP, however, homomeric, Q/R edited GluR2 (GluR2(R)) largely remains within the ER. The positively charged arginine residue introduced by editing, is crucial in conferring calcium-impermeability on GluR2-containing AMPARs, but also appears to act as an ER retention signal (Greger et al., 2002; Greger and Esteban, 2007). Thus, GluR2 subunits not co-assembled with unedited (glutamine, at the Q/R site) subunits, are held within the ER (Greger et al., 2002, 2003).

There is compelling evidence, derived from a variety of experimental approaches, to support the view that the initial interaction between TARPs and AMPARs occurs at the level of ER membrane. First, co-expression of GluR2(R) with stargazin appears to reduce the barrier to ER export, allowing a significant increase in the expression of homomeric GluR2 receptors at the cell surface (Yamazaki et al., 2004). Stargazin similarly enhances the trafficking and expression of flop AMPAR isoforms, which are normally exported from the ER less well than the flip variants (Coleman et al., 2006). Second, FRET studies have shown that YFP tagged AMPARs quench the fluorescence of CFP tagged stargazin molecules within the ER (Bedoukian et al., 2006). Third, the ER chaperone BiP (Ig binding protein), appears to be involved in the interaction between AMPARs subunits and TARPs (Vandenberghe et al., 2005b) reinforcing the view that TARPs associate with AMPARs in the ER membrane (Fig. 1).

As AMPARs mature and pass from the ER through the trans-Golgi network (TGN), they undergo additional post-translational modifications—predominantly glycosylation and phosphorylation—before being packaged into vesicles for cell surface delivery. TGN processing and post-translational modification commonly make significant additional contributions to the molecular weight of the mature protein. Thus, when these modifications are removed, the residual change in molecular weight can be used as an indicator of AMPAR maturation (Greger et al., 2002). Co-expression of stargazin in cerebellar GCs from *stargazer* mice, increases the proportion of AMPARs that display the heavy molecular weight of the mature glycosylated protein, supporting the view that TARPs assist AMPAR trafficking through the Golgi apparatus, and beyond, to the plasma membrane (Tomita et al., 2003) (Fig. 1).

Various secondary proteins, which interact with the TARPs, have been implicated as mediators of this Golgi and membrane trafficking. These include nPIST (neuronal isoform of protein-interacting specifically with TC10; Cuadra et al., 2004), map 1 LC2 (microtubule-associated protein 1 light chain 2; Ives et al., 2004), MAGI 2 (membrane-associated guanylate kinase, WW and PDZ domain containing 2; Deng et al., 2006) and the adapter protein complex AP-4 (Matsuda et al., 2008) (Fig. 1). The *C-*terminal of the TARP protein mediates these interactions, and fusion of the stargazin C-terminus to the AMPAR facilitates membrane trafficking (Bedoukian et al., 2008).

TARPs assist AMPAR delivery specifically to the synaptic membrane (Chen et al., 2000; Bats et al., 2007). While AMPARs that are co-assembled with stargazin can diffuse rapidly and freely in the cell surface, the PDZ-binding domains of the TARP can interact with PSD-95 in the postsynaptic density, causing AMPARs to accumulate at synaptic sites (Bats et al., 2007). As expected, truncated stargazin that lacks this anchoring region (StargazinΔC), fails to rescue synaptic AMPAR responses in *stargazer* GCs (Chen et al., 2000). There also is growing evidence for an involvement of TARPs in synaptic plasticity, specifically in the bidirectional control of hippocampal LTP (Tomita et al., 2005a). Nine serine residues present in the C-tail of stargazin (and of γ-3, -4 and -8) are phosphorylated by CaMKII and PKC. This allows enhanced movement of AMPARs (Tomita et al., 2005a), which can be suppressed by de-phosphorylation via PP1. Although the mechanism is poorly understood, it is suggested that serine phosphorylation neutralizes the charge on nearby basic residues, decreasing their interaction with negatively charged membrane phospholipids and thus increasing AMPAR membrane mobility (Tomita et al., 2005a). Subsequent TARP interaction with PSD-95 would immobilize and anchor the AMPAR at the synapse. While it remains unclear how TARP de-phosphorylation by PP1 would result in a loss of synaptic AMPARs, phosphorylation by PKA disrupts the PDZ-binding site of the TARPs and is expected to facilitate AMPAR removal from synapses (Chetkovich et al., 2002; Choi et al., 2002).

## TARPs are “intrusive” chaperones that modify surface AMPAR properties

**Fig. 2:**
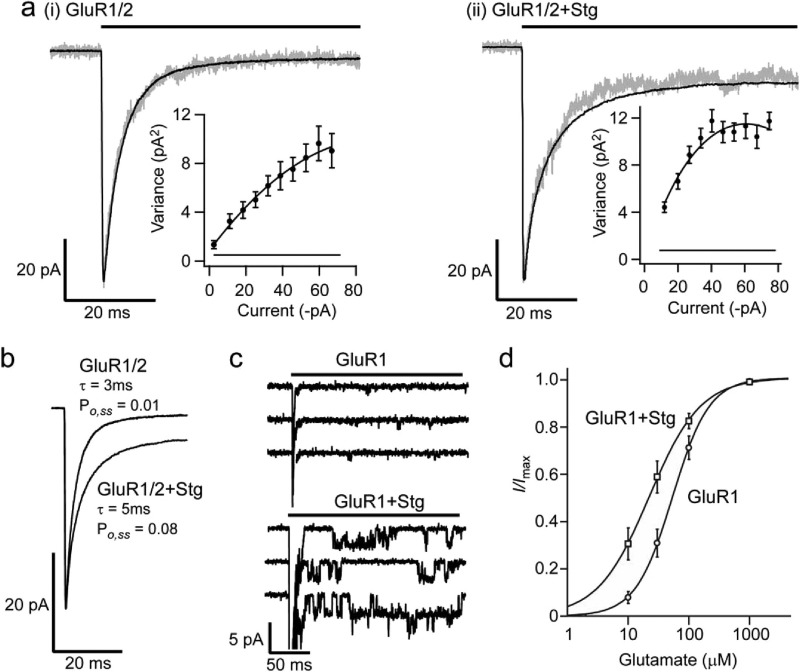
Stargazin alters AMPAR properties. (a) (I) (ii), Currents evoked at −60 mV by rapid application of 10 mM glutamate (200 ms) to outside-out patches from cells expressing heteromeric GluR1/2 AMPARs in the absence and presence of stargazin (Stg). Black lines show means of 84 or 98 traces respectively; grey lines show representative traces. Inset shows a plot of variance versus mean current, obtained from non-stationary fluctuation analysis. Baseline variance is also displayed. For these cells the slope of the relationship gave a weighted mean single–channel conductance of 3.5 pS in the absence of Stg, and 6.5 pS in its presence. Thus, Stg associated AMPARs show an increased conductance and peak open probability (for methodology see Soto et al., 2007). (b) Current traces from a (i), (ii), super imposed and shown on an expanded time base (AMPAR currents with and without Stg are indicated), to illustrate the slower desensitization kinetics and larger steady-state open probability of receptors expressed with Stg. (c) Resolved single channel currents from homomeric GluR1 AMPARs expressed in the absence (upper traces) and presence (lower traces) of Stg, illustrating the increased single-channel conductance, slower kinetics and higher open probability of AMPAR expressed with a TARP. Each set of recordings shows three responses to fast glutamate application. Initial truncated peaks are followed by clear single-channel currents in the presence of Stg, and by openings that are barely discernible in its absence. (d) Dose–response curves of peak AMPAR current amplitude illustrating that that Stg increases the glutamate affinity of GluR1 (a–c unpublished observations; d, from Tomita et al., 2005b).

In addition to their role in AMPAR trafficking TARPs remain bound to, and modify the functional properties of, AMPARs present in the cell membrane (Kristensen and Traynelis, 2005). The TARPs increase the single-channel conductance of AMPARs by between 40 and 130% (Fig. 2A, C;Tomita et al., 2005b; Soto et al., 2007, 2009). This, together with the fact that TARPs slow the deactivation and desensitization kinetics (Tomita et al., 2005b; Priel et al., 2005; Cho et al., 2007; Korber et al., 2007) greatly enhances charge transfer through TARP-associated AMPAR channels (Fig. 2B, C). These kinetic changes, which increase the steady-state current mediated by AMPARs (Priel et al., 2005; Turetsky et al., 2005), also slow the time course of the synaptic current (Milstein et al., 2007), and likely enhance currents generated during transmitter spillover.

TARPs also modify certain of the basic pharmacological properties of AMPARs. They increase the apparent glutamate affinity of AMPARs by roughly threefold (Fig. 2D; Tomita et al., 2005b; Turetsky et al., 2005), while decreasing the affinity of CP-AMPAR channels for intracellular spermine (Soto et al., 2007). Furthermore, the presence of a TARP modifies the action of CNQX which is a classical competitive AMPAR *antagonist* on “TARPless” AMPARs, but is converted to a *partial agonist* when AMPARs are co-assembled with a TARP (Menuz et al., 2007). This change in AMPAR pharmacology is suggested to depend, at least in part, on the extracellular loop between M1 and M2 (Ex1) of the TARP molecule. This region appears to be one of the primary sites through which TARPs interact with AMPAR subunits (Tomita et al., 2007).

One particularly intriguing question, concerning AMPAR-TARP interaction in the cell surface, is whether this is under any form of functional control. Clearly a mechanism that caused dissociation of TARPs from AMPARs could trigger a rapid change in AMPAR properties. Indeed it has recently been demonstrated that transmitter activation may instigate dissociation of AMPARs from stargazin, and lead to AMPAR endocytosis (Tomita et al., 2004; Morimoto-Tomita et al., 2009). In the short term, dissociation of the TARP would be expected to cause a decrease in the current generated by AMPARs, due to the reduction in charge transfer. However, more subtle changes could also occur, such as increased sensitivity to intracellular polyamine block at synapses which express CP-AMPARs.

The presence of various protein partners and trafficking molecules that interact with AMPARs at synapses, could suggest that TARPs are not always essential for AMPAR trafficking. Proteins such as PICK1 (Xia et al., 1999), GRIP1 (Dong et al., 1997) and ABP (Srivastava et al., 1998) have been suggested to fine tune synaptic receptor delivery and trafficking in the absence of any other chaperone molecules (Ziff, 2007). However, there is compelling evidence that TARP-associated AMPARs far outnumber those AMPARs that are combined with other protein partners (Fukata et al., 2005). It is possible that, while TARPs traffic receptors to the synapse and remain bound to surface receptors, the TARP dissociates prior to AMPAR endocytosis. Such AMPARs may then either be degraded or recycled by other protein partners such as PICK1 or GRIP1 (Ziff, 2007).

## Distribution of TARP family members in cerebellar neurons and glia

**Fig. 3:**
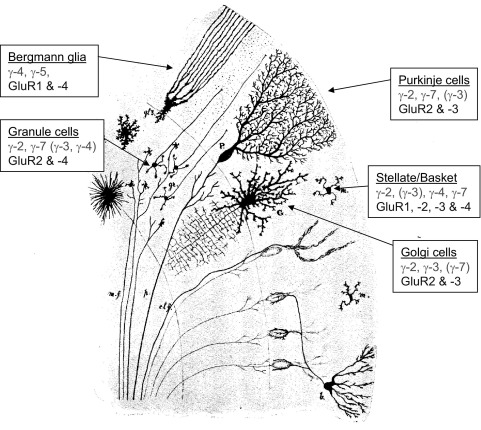
TARP (γ−) subtype expression in cerebellar cell types. Main TARPs have been identified by rt-PCR, immunocytochemistry and *in situ* hybridization. Main AMPAR subunits (GluRs) are also indicated (see text). The differential distribution of TARPs, together with AMPAR subunit diversity, provides a rich variety of functionally distinct AMPAR subtypes in different cell types (Tomita et al., 2003; Fukaya et al., 2005; drawing of cerebellum after Sharpey-Schafer, 1929).

TARPs are expressed differentially with respect to brain region and ontogenic period (Fukaya et al., 2005), greatly increasing the potential for AMPAR diversity tailored to the needs of particular pathways. γ-4 Is prominent throughout the brain in early development (Tomita et al., 2003; Fukaya et al., 2005). At later stages, stargazin and γ-7 are intensely expressed in the cerebellum (Tomita et al., 2003; Fukaya et al., 2005; Kato et al., 2007); γ-8 is most pronounced in the hippocampus (Tomita et al., 2003; Fukaya et al., 2005); γ-3 is highest in the cerebral cortex (Tomita et al., 2003; Fukaya et al., 2005); while γ-5 is most noticeable in the olfactory bulb (Fukaya et al., 2005). All TARPs appear to be represented within the cerebellum, although to varying extents in different cell types (Fig. 3). Thus from immunocytochemistry (Tomita et al., 2003; Kato et al., 2007) and *in situ* hybridization studies (Tomita et al., 2003; Fukaya et al., 2005; Lein et al., 2007) cerebellar GCs express stargazin. Perhaps less expected, they also appear to express low levels of γ-3, γ-4 and γ-7 (Fukaya et al., 2005), which might explain why small AMPAR responses are present in cultured *stargazer* GCs (Kato et al., 2007). Molecular layer interneurons (basket cells and stellate cells) express various levels of stargazin (γ-2), γ-3, γ-4 and γ-8 (Fukaya et al., 2005) while Golgi cells express γ-2, γ-3 and γ-7 (Menuz et al., 2008). Bergmann glia (BG) express γ-4 and γ-5 (Tomita et al., 2003; Fukaya et al., 2005; Lein et al., 2007) while cerebellar astrocytes express γ-4 (Tomita et al., 2003). Purkinje cells express γ-2 and γ-7 (Tomita et al., 2003; Fukaya et al., 2005; Kato et al., 2007; Lein et al., 2007). In the next section we will consider the TARPs and AMPAR subunits expressed in each of these different cerebellar cell types, in relation to functional aspects of the individual cells.

## Cerebellar granule cells

Cerebellar GCs have been crucial to the discovery that TARPs play a critical role in central synaptic transmission. GCs appear to be relatively unusual in that delivery of their synaptic AMPARs seems dependent on a single type of TARP (γ-2), that is lost in a naturally occurring mutant mouse—*stargazer* (Fukaya, 2005). This naturally occurring recessive mutant (Noebels et al., 1990) has a range of neurological phenotypes including ataxic gait, absence seizures (involving an upwards stare, hence stargazer), lack of eye-blink conditioning, small size and a decreased Mendelian ratio—indicating higher rates of gestational mortality. The GCs of this mouse are characterized by a striking lack of synaptic AMPAR-EPSC at the mossy fibre-GC synapse (Chen et al., 2000).

Since the native AMPARs in GCs can reach the surface membrane only when successfully associated with an exogenous TARP, the recovery of AMPAR responses in cultured *stargazer* GCs transfected with potential TARPs, has provided a simple experimental system for identifying members of the stargazin family of proteins as being indisputable TARPs (Tomita et al., 2003; Kato et al., 2007). Cerebellar GCs express GluR2 and GluR4 AMPAR subunits, with flop isoforms predominating in older cells (Mosbacher et al., 1994; Bahn and Wisden, 1997). The GluR4 subunit in these cells exists in its uncommon short form (GluR4c), which possesses an alternatively spliced C-tail (Gallo et al., 1992). The fact that the two main receptor subunits in GCs both have short C-tails could explain why subunit selective TARPs might appear ineffective at AMPAR trafficking in these cells (Kato et al., 2007, 2008).

There is compelling evidence for a direct association of TARPs with AMPARs in GCs. Experiments using nondenaturing PAGE have demonstrated that AMPARs obtained from “wild type” GCs migrate in a large TARP-containing complex. In contrast AMPARs from *stargazer* GCs lack the associated TARP (Vandenberghe et al., 2005a). Recent experiments suggest that the properties of GC AMPARs may vary with the level of TARP present, supporting the idea that the AMPAR-TARP complex shows variable stoichiometry (Milstein et al., 2007).

Although studies of GCs from *stargazer* mice have provided considerable information about TARPs control of AMPAR function, they have also highlighted a lack of basic knowledge about synaptic development and plasticity in these cells.

Unlike AMPARs in many central neurons, those in GCs are highly localized in the postsynaptic membrane at all ages. Few, if any, AMPARs can be detected in extrasynaptic patches (DiGregorio et al., 2002; Cathala et al., 2005). It remains unclear whether this unusual arrangement reflects a specific property of stargazin or its associated cytoskeletal molecules in GCs, and therefore whether the AMPAR-stargazin assembly is able to move freely in the extrasynaptic membrane, as it does in other cell types (Bats et al., 2007). Interestingly, the presence of postsynaptic NMDAR-dependent LTP at mossy fibre-GC synapses (Gall et al., 2005; Mapelli and D'Angelo, 2007), would suggest that AMPARs are relatively mobile in the surface membrane of GCs.

## Cerebellar stellate and basket cells

In addition to their effects on AMPAR transport, gating and desensitization, it has recently been shown that the TARPs also modify the block of CP-AMPARs by intracellular polyamines. Cerebellar interneurons show inwardly rectifying synaptic currents at the parallel fibre–stellate cell (PF-SC) synapse (Liu and Cull-Candy, 2000), indicative of the presence of GluR2-lacking calcium permeable (CP) AMPARs. Despite the presence of GluR2-lacking synaptic AMPARs, these cells are thought to express GluR2, along with GluR1, -3 and -4 AMPAR subunits (Keinanen et al., 1990; Petralia et al., 1997; Gardner et al., 2005; Rossi et al., 2008), and γ-2, γ-4, and γ-7 (possibly, with low levels of γ-3) TARP family members (Tomita et al., 2003; Fukaya et al., 2005). The inward rectification of CP-AMPARs is conferred by endogenous intracellular polyamines which plug the CP-AMPAR channel at depolarized potentials (Bowie and Mayer, 1995; Kamboj et al., 1995; Koh et al., 1995). It has recently been shown that blocking of these AMPAR channels by intracellular polyamines is attenuated by coassembly with a TARP in recombinant systems, thus enhancing the charge transfer mediated by CP-AMPARs (Soto et al., 2007).

This is of particular relevance in cerebellar stellate cells, as these cells display a novel form of synaptic plasticity, characterized by a rapid and lasting change in the subunit composition and Ca^2+^ permeability of their synaptic AMPARs (Liu and Cull-Candy, 2000, 2005; Gardner et al., 2005). The rapid change in AMPAR subunit composition can be readily identified as a reduction in the rectification of the I–V relationship when CP-AMPARs are replaced by CI-AMPARs. A qualitatively similar but more gradual reduction in CP-AMPARs also occurs during development (Soto et al., 2007).

There is functional evidence to suggest that both CP- and CI-AMPARs are associated with a TARP in stellate cells (Soto et al., 2007). Thus, experiments have been carried out to compare the rectification index (RI) and single-channel conductance of recombinant AMPAR channels with those channels that underlie the AMPAR-mediated EPSCs in these cells. The properties of recombinant homomeric GluR3-containing CP-AMPARs, and heteromeric GluR2/3 CI-AMPARs, display properties that match well to those of rectifying (CP−) and non-rectifying (CI−) AMPAR-EPSCs, when the recombinant receptors are co-assembled with a TARP. It seems likely that the rapid replacement of CP- by CI-AMPARs in stellate cells will depend on the presence of specific TARPs, although this remains to be seen.

It is of interest that PF-SC synapses display glutamate spillover during physiologically relevant high frequency activity (Carter and Regehr, 2000). It is possible that the decrease in densensitization, and increase in steady-state current, conferred on AMPAR by the presence of a TARP, could enhance such spillover currents. Furthermore, glutamate spillover has been identified at inhibitory basket/stellate cell–PC synapses where it activates presynaptic AMPARs (Satake et al., 2000; Rusakov et al., 2005; Liu, 2007). It is currently unknown whether AMPARs present in the axon or the presynaptic terminal are associated with TARPs, and if so how the TARP contributes to this non-dendritic protein trafficking.

## Golgi cells

Cerebellar Golgi cells are known to express γ-2, γ-3 and γ-7. AMPARs in these cells appear largely unaffected in *stargazer* mice, and are also unaltered in a γ-3^−/−^ knockout mutant mouse. Thus, either TARP alone is sufficient to maintain normal synaptic function and AMPAR kinetics, suggesting that TARP family members may compensate for the loss of other TARPs and that this TARP “redundancy,” in many neurons, may explain why loss of a single TARP often fails to produce a behavioral phenotype (Menuz et al., 2008). However, Golgi cells in mice in which both γ-2 and γ-3 have been deleted, contain very few AMPARs. Additionally, it is of note that Golgi cells from the double knockout exhibit more rectifying (GluR2-lacking) synaptic responses, suggesting that the type of TARP present (in this case γ-7) will influence the subunit composition of the synaptic receptors.

## Cerebellar glia

It is well established that many glial cell types express functional AMPARs (Wyllie et al., 1991; Burnashev et al., 1992; Lin et al., 2005; Gallo, 2007). In oligodendrocytes, their progenitor cells (NG2^+^ cells—one of the cell types involved in generating oligodendrocytes throughout life) and BG, CP-AMPARs appear to be the predominant subtype present (Burnashev et al., 1992; Iino et al., 2001; Bergles et al., 1997; see Gallo, 2007). Both cerebellar astrocytes (Tomita et al., 2003) and BG express γ-4 (Tomita et al., 2003; Fukaya et al., 2005; though see Lein et al., 2007) with, BG cells additionally expressing the TARP γ-5 (Fukaya et al., 2005). While TARPs in neurons retain AMPARs at the synapse by attaching to PSD-95 (Bats et al., 2007), in glia that lack localized PSD-95 the function of TARPs remains enigmatic (see Soto et al., 2009). However, it is of note that TARPs also interact with other MAGUKs, including SAP-97, PSD-93 and SAP-102 (Dakoji et al., 2003; Ives et al., 2004), which do appear to be present in glia (Claudepierre et al., 2000; Leonoudakis et al., 2001; Douyard et al., 2007).

It is clear that AMPAR are activated by neurally released transmitter in a number of glia. Indeed, it has been demonstrated in the cerebellum that climbing fibres make direct synaptic contact onto NG^2+^ cell processes at anatomically defined synaptic junctions, where transmitter release activates CP-AMPARs (Lin et al., 2005). Furthermore, functional axon–glial synapses have recently been identified on local oligodendrocyte precursor cells in the corpus callosum (Kukley et al., 2007; Ziskin et al., 2007; see Gallo, 2007), while in BG, the CP-AMPARs (Burnashev et al., 1992; Wisden and Seeburg, 1993) are activated by ectopic release of transmitter from climbing fibres and by transmitter spillover (Bergles et al., 1997; Dzubay and Jahr, 1999; Bergles et al., 2000; Matsui and Jahr, 2003, 2004; Matsui et al., 2005; Lin et al., 2005; Piet and Jahr, 2007). This activation of CP-AMPARs in BG plays an essential part in the glial ensheathment of Purkinje cell synapses that is required for these synapses to function normally (Iino et al., 2001; Watanabe, 2002). Indeed, in BG cells that have been virally transfected with GluR2, so that AMPARs lose their calcium permeability, BGCs cease to perform their ensheathing behavior that is required for rapid clearance of the synaptic transmitter (Iino et al., 2001). It has recently been shown that SAP-97 is expressed at perisynaptic sites in BGs, and could thus provide an anchor allowing TARP-associated CP-AMPARs to accumulate (Douyard et al., 2007).

Additionally, the TARPs display cell–cell adhesion behavior, similar to that of their protein ancestors the claudins. This seems to involve direct protein contact between TARPs in tightly apposed cells (Price et al., 2005). Such interactions may also prove vital in neuronal–glial association.

## Purkinje cells

Cerebellar Purkinje cells express γ-2 and γ-7 (Fukaya et al., 2005; Lein et al., 2007; Menuz and Nicoll, 2008), as well as heteromeric GluR2/3 containing AMPARs (Lambolez et al., 1992; Momiyama et al., 2003). Unlike the Golgi cells, where deletion of γ-2 produced little change in synaptic properties, Purkinje cells from *stargazer* mice exhibited a marked decrease in the size and observed frequency of mEPSCs despite the continued high expression of the TARP γ-7 (Menuz and Nicoll, 2008). This observation provides evidence that γ-7 is much less effective, than other TARPs, in delivering AMPARs to synaptic sites.

## Conclusion

Much initial information, concerning the importance of TARPs in dictating AMPAR function, has arisen from experiments which sought to identify the cause of the defect in the mutant mouse *stargazer*. Because this defect primarily appeared to affect cerebellar GCs, these have been an initial focus of attention in many experiments. Although it is now clear that TARPs control AMPAR properties throughout the CNS, the cerebellum continues to be one of the preferred brain region for studies involving this important family of proteins. An unusual combination of factors makes cerebellar GCs in the *stargazer* mouse of particularly experimental value: (1) GCs can be readily identified and maintained in primary culture. (2) In wild-type mice these cells express mainly stargazin; although low very levels of other TARPs are probably also present, these do not “rescue” surface AMPAR expression. (3) As GCs from *stargazer* mice lack detectable surface AMPARs, cultured GCs are a convenient system in which to examine and test other potential TARPs (identified from their ability to rescue AMPAR responses), and in which to determine efficacy of chimeric TARPs. It is also of note that most, if not all, members of the TARP family are represented within the various cell types present in the cerebellum. This adds further to the value of the cerebellum in yielding clues about the way in which particular TARPs and AMPARs subtypes are tailored to match the needs of particular pathways, synapses and neuronal–glial connections.
